# Support Community Formation on a Mobile App for People Living With HIV and Substance Use Disorder: A Computer-Mediated Discourse Analysis

**DOI:** 10.2196/66564

**Published:** 2026-01-15

**Authors:** Adati Tarfa, Kristen Pecanac, Olayinka Shiyanbola, Cameron Liebert, Sarah Dietz, Rebecca Miller, Ryan P Westergaard

**Affiliations:** 1 Yale School of Medicine, New Haven, CT, United States; 2 School of Nursing, University of Wisconsin-Madison, Madison, WI, United States; 3 College of Pharmacy, University of Michigan, Ann Arbor, MI, United States; 4 School of Medicine and Public Health, University of Wisconsin-Madison, 610 Walnut Street, Madison, WI, 53726, United States, 1 6082632880

**Keywords:** HIV, mobile app, social support, computer-mediated, discourse analysis, virtual community, people living with HIV, substance use disorders, SUD, online support, online communities, mHealth, mobile health, HIV care, opioid use disorder, qualitative analysis, OUD

## Abstract

**Background:**

People living with HIV and substance use disorders (SUDs) have complex health care needs requiring adaptive and effective support systems. While mobile health apps can foster virtual communities grounded in shared lived experiences, little is known about the dynamics within these digital spaces.

**Objective:**

We examined the formation of a virtual community on the Addiction Comprehensive Health Enhancement Support System (A-CHESS; The Center for Health Enhancement Systems Studies, University of Wisconsin-Madison College of Engineering) message board, a mobile app designed to support HIV care engagement among individuals living with HIV and SUD.

**Methods:**

We conducted a computer-mediated discourse analysis of A-CHESS message board posts to examine communication patterns, interaction structures, and engagement dynamics. Quantitative comparisons were used to assess differences between posters and nonposters using *t* tests and chi-square tests. We then applied qualitative coding to categorize messages by type, speaker, and function to understand how staff and participants coconstructed a supportive virtual environment.

**Results:**

Among 208 participants, 87 (42%) posted at least once on the A-CHESS message board, contributing 1834 messages between April 2019 and May 2021. Posters and nonposters did not differ significantly in age (*t*_206_=–0.64; *P*=.52), gender (*χ*²_1_=0.14; *P*=.71), or race (*χ*²_1_=0.52; *P*=.47). We identified 3 message types: premeditated, adlib, and participant-driven. Staff initially led with premeditated messages (eg, recovery stories, HIV risk information, and “Thought of the Day” inspiration), which participants often interpreted and adapted to their own SUD recovery. Over time, staff incorporated adlib messaging styles using personalized narratives and polls to sustain engagement. Participants then developed their own posts using similar formats, incorporating Alcoholics Anonymous literature, sharing legal and personal challenges, and suggesting new app features (eg, medication check-ins to support adherence).

**Conclusions:**

A-CHESS staff adapted communication styles to increase engagement, while participants appropriated the app’s message board to reflect personal goals and lived experiences. Mobile health interventions may benefit from design elements that support participant-led discourse and customization, fostering ownership, support, and relevance within virtual care communities.

## Introduction

The HIV infection is highly prevalent among people who use drugs and alcohol. Among an estimated 8.9 to 22.4 million people who inject drugs globally, approximately 0.9 to 4.8 million are HIV positive [1]. Additionally, alcohol dependence is found in 40% to 50% of individuals living with HIV, and rates of cannabis and stimulant use are also higher among people living with HIV compared to the general population [2,3]. Individuals with comorbid HIV and substance use disorder (SUD) also experience a lower quality of life, with higher rates of comorbidities, psychological distress, and social stigma than those living with HIV alone [4-6]. With the widespread availability of mobile phones, individuals living with SUD and HIV can access social support through mobile health apps [7]. Message boards within these mobile health interventions are recognized as critical sources of social support for people living with HIV, facilitating communication and support among community members [8].

Despite recognizing message boards’ importance and widespread usage, only a few studies have explored the dynamics within these boards to optimize participant engagement and enhance social support. Previous studies have mainly focused on the feasibility [9], frequency [10], and types of messages posted on the message boards [11,12] and the characteristics of individuals posting [13,14]. Further examination is needed to explore how a supportive community is formed within these apps, especially for the key population of individuals with comorbid HIV and SUD.

To address the research gap, our study used discourse analysis to examine the formation of virtual communities that offer social support for people living with HIV and SUD. Discourse analysis explores natural talk and texts, revealing how language patterns and practices shape society and individuals [15]. Previous studies have used discourse analysis in HIV research to explore communication on HIV informative posters regarding treatment adherence and the prevention of stigmatization [16]. Discourse analysis has also been used to understand the behaviors of men who use drugs concerning viral load and reliance on antiretroviral therapy during sexual encounters [17]. However, discourse analysis has not yet been applied to explore virtual support communities of individuals living with HIV and SUD in the context of social communities.

Our study used computer-mediated discourse analysis [18] to explore the community message board feature of the Addiction Comprehensive Health Enhancement Support System (A-CHESS; The Center for Health Enhancement Systems Studies, University of Wisconsin-Madison College of Engineering), a mobile health app designed to provide social support and improve engagement in HIV care for individuals living with HIV and SUD [19].

## Methods

### A-CHESS App Overview

A-CHESS is a modified version of the evidence-based CHESS, which has proven efficacy in preventing relapse among individuals with alcohol use disorder [20]. A-CHESS was adapted for use among people living with SUD and HIV by expanding additional features aimed at promoting medication adherence, facilitating regular treatment evaluations, providing access to a community message board, offering case management services, and conducting weekly surveys to monitor recovery progress and evaluating protective and risk factors in the social environment [19]. The A-CHESS app was based on Self-Determination Theory, emphasizing competence, autonomy, and social relatedness to motivate participants to engage in healthy behaviors [21].

### Setting and Participant Recruitment

Participants were recruited through the distribution of recruitment flyers at HIV clinics and collaboration with case managers and physicians for referrals. Inclusion criteria required participants to be 18 or older, have a documented HIV infection, be able to speak and write in English, and have a history of SUD. SUD was determined based on positive screening results, current engagement in substance use treatment, a lifetime history of problematic drug or alcohol use, or recent substance use–related incarceration. Enrollment occurred on a rolling basis from January 2019 to April 2019.

Eligible participants were instructed to download the A-CHESS app onto their smartphones. Case managers provided tutorials to guide participants through the process of using the app, including creating a deidentified username to protect their offline identities and ensure anonymity. Participants were then familiarized with the app’s various features, receiving step-by-step instructions on navigating and using its functionalities.

### Data Source

The primary data source for our study was the A-CHESS message board, a public forum within the A-CHESS app where participants could engage with case managers, research staff, and peers. Our analysis included all messages posted by the participants and messages from the research staff and case managers, recognizing their membership in the virtual community and their contributions to community formation [22]. Participants were provided with guidelines on appropriate message board use and respectful dialogue. Participants were instructed not to share personally identifying information on the message board, and informed that their posts were immediately visible to other users. The study team monitored the message board content, and personal contact through phone or text was available. In addition to the message board data, we collected baseline survey information to describe participants’ demographics, history of HIV, history of SUD, and substance use patterns.

### Data Sampling

To conduct a longitudinal examination of virtual community formation, we analyzed all the messages posted on the message board throughout the 26-month study period. Research staff extracted the information from the message board, including the posted messages, usernames, and time of posting for the analysis. Each message board posting or comment was placed into separate cells within an Excel (Microsoft) workbook. The unit of analysis was an individual message posted. When examining data in a virtual community, various approaches can be used, such as random selection of messages, analyzing messages by theme (eg, all messages in a particular thread), focusing on specific time frames (eg, messages from a particular week), studying specific phenomena (eg, messages related to jokes or conflict negotiation), or analyzing messages by group (eg, messages posted by women or individuals who identified a particular substance as their drug of choice) [18,23]. Our sampling strategy combined sampling based on time and theme. Initially, we organized all the messages posted on the message board based on their respective time stamps, arranging them chronologically from the first to the last post on the message board. We refined our data management approach by reorganizing the messages based on the threads they belonged to, considering the corresponding time. For example, if a thread had an initiation message posted in May 2019 and a response message posted in December 2019, we analyzed the May and December messages together as they formed part of the same thread.

### Data Analysis

For the demographic data, we conducted independent samples *t* tests and chi-square tests to examine differences between posters (participants who posted at least once on the A-CHESS message board) and nonposters. Age was summarized using means and SDs; categorical variables were summarized using counts and percentages. A significance level of *P*<.05 was used.

The authors used a computer-mediated discourse analysis (CMDA) approach to understand how a virtual community is being formed on the A-CHESS message board [23]. CMDA analyzes language use in online communication, including social media, chat rooms, and email by examining the linguistic features, communication strategies, and interaction patterns of the communication. CMDA additionally examines social dynamics in online communication and how their structure reveals social behaviors. According to Herring [18,23,24], virtual communities are formed when individuals use computer-mediated platforms, such as A-CHESS, by establishing structure, deriving meaning in their communication, interacting, and engaging in social behaviors within these virtual communities.

#### Quantitative Analysis: Structure and Interaction of A-CHESS Virtual Community Formation

To explore the structure and interaction patterns of the virtual community, we conducted a quantitative analysis of user interactions on the A-CHESS message board. This involved quantifying message types, examining discourse structure, and describing the participants’ demographics in virtual community formation. Through this analysis, we identified patterns in language use, key participants, reciprocity of engagement, and the occurrence of extended conversations. Descriptive statistics were performed using SPSS (v28; IBM Corp) to describe the study participants’ characteristics.

#### Qualitative Analysis: Meaning, Social Behaviors, and Engagement Patterns of the Discourses in the Message Board

The qualitative analysis explored how participants constructed meaning in their interactions and the language used to indicate their social behavior. This included examining participants’ responses to messages, the types of messaging they initiated, and the level of participation exhibited by community members. By focusing on these aspects, we gained valuable insights into the evolving nature of the app discourse. We explored the range of topics discussed and how participants engaged with, responded to, and shaped the overall patterns of interaction.

#### Coding Process and Framework

The coding framework used in this study was developed by the researchers (AT and KP) to capture the various aspects of the messages, including their structure, meaning, interaction, and social behaviors. AT, who has received training in qualitative research methods, thoroughly reviewed the messages over 2 months. Following an inductive approach, the researchers examined the patterns of interaction on the message board. Initially, a quarter of the data were coded sequentially, with extensive discussions held for each message to reach a consensus on the assigned codes. These codes were then applied to the remaining data. Each message was assigned one or more codes to capture specific actions and behaviors. The researchers categorized these behaviors based on the structure of the messages, identifying common themes and patterns. Additionally, the messages were grouped according to the key participants involved, focusing on understanding the actors within the interactions.

To ensure rigor and trustworthiness, we drew on Lincoln and Guba criteria of credibility, transferability, dependability, and confirmability [25]. Credibility was enhanced through prolonged engagement with the data, iterative coding discussions between 2 trained researchers, and the lead analyst’s (AT) sustained involvement in the HIV community. AT and KP developed codes inductively, refining them through joint review and consensus. Transferability was supported by providing rich contextual detail on the A-CHESS platform, participant characteristics, and the broader setting of HIV and substance use recovery, along with representative excerpts in the results. Dependability was addressed through consistent documentation of coding decisions, a staged and collaborative coding process, and an audit trail of coded data. Confirmability was strengthened through analytic transparency, discussion of researcher positionality, and linking interpretations to both direct participant excerpts and the guiding discourse analytic framework. To further promote transparency, the manuscript follows the Standards for Reporting Qualitative Research (SRQR) [26]; the completed checklist is provided in Checklist 1.

#### Reflexivity Statement

Qualitative researchers AT and KP bring complementary clinical and methodological expertise to this analysis. AT is a pharmacist with PhD training in health services research, and KP is a nurse and PhD-trained Associate Professor of Nursing who teaches advanced qualitative research methods. Both researchers have cared for people living with HIV in their clinical roles. AT has also developed relationships with members of the HIV community outside research through volunteer participation in community-based social events. Additionally, AT is trained under the team that developed A-CHESS and has been involved in the app’s redesign, which informs a deep familiarity with the platform’s context and use.

### Ethical Considerations

This study received ethical approval from the University of Wisconsin–Madison Institutional Review Board (2016-1190). All participants provided informed consent at enrollment, including agreement for the use of their deidentified app data for research purposes. To protect privacy and confidentiality, participants used pseudonymous usernames on the A-CHESS message board, and all data were deidentified prior to analysis. No identifiable personal health information was included in the dataset. Participants were compensated for completing weekly surveys and milestone activities within the app, while they were not compensated for posting on the message board.

## Results

### Overview

Between March 13, 2019, and April 30, 2021, a total of 2071 messages were shared on the A-CHESS message board by research staff (including case managers) and study participants. Blank and duplicate posts were excluded from the analysis, resulting in 1834 posts being included in the discourse analysis. All 208 study participants were provided access to the A-CHESS message board and information on how to post to the message board. Approximately 42% (87 individuals) of all study participants posted on the message board at least once. The distribution of age, race, sex, employment, and education was similar for A-CHESS study participants who actively engaged with the message board (n=88), and those who did not post at all on the message board (n=120) are shown in Table 1.

**Table 1.** Demographic characteristics of participants who posted on the message board vs. nonusers.^a^

Variable	Message board users (n=88)	Message board nonusers (n=120)
Mean (SD), age (years)	45.88 (11.5)	46.69 (11.0)
Sex, n (%)		
Male	65 (74.7)	91 (75.8)
Female	22 (25.3)	26 (21.7)
Other	1 (1.1)	—^b^
Race, n (%)		
Black	57 (64.8)	80 (66.7)
Education, n (%)		
Four-year college	31 (35.2)	45 (37.5)
Employment, n (%)		
Yes	54 (62.1)	85 (70.8)
No	34 (39.0)	35 (29.2)

a Values are presented as mean (SD) for continuous variables and number (percentage) for categorical variables. Percentages may not total 100% due to rounding. Only selected categories are shown.

b Not available.

Posters and nonposters did not differ significantly in age, *t*_206_=–0.64, *P*=.52; gender, *χ*²_1_=0.14, *P*=.71 (n=210); or race, *χ*²_1_=0.52, *P*=.47 (n=197).

Out of the 1834 messages posted on the message board, the majority (n=1,283, 70%) were contributed by study participants, while the remaining 30% (n=550) were posted by research staff members, including 2 case managers. A small subset of participants played a significant role in contributing to the engagement on the message board. Specifically, 5 participants were responsible for 40% (n=733) of all the posts, with one alone accounting for 12% (n=220) of the messages. A significant portion (n=62, 70%) of the participants posted between 1 to 10 messages. Less than 22% (n=403)of the posts were single posts without further engagement.

### Meaning, Social Behaviors, and Engagement Patterns of the Discourses in the Message Board

**Figure 1.**
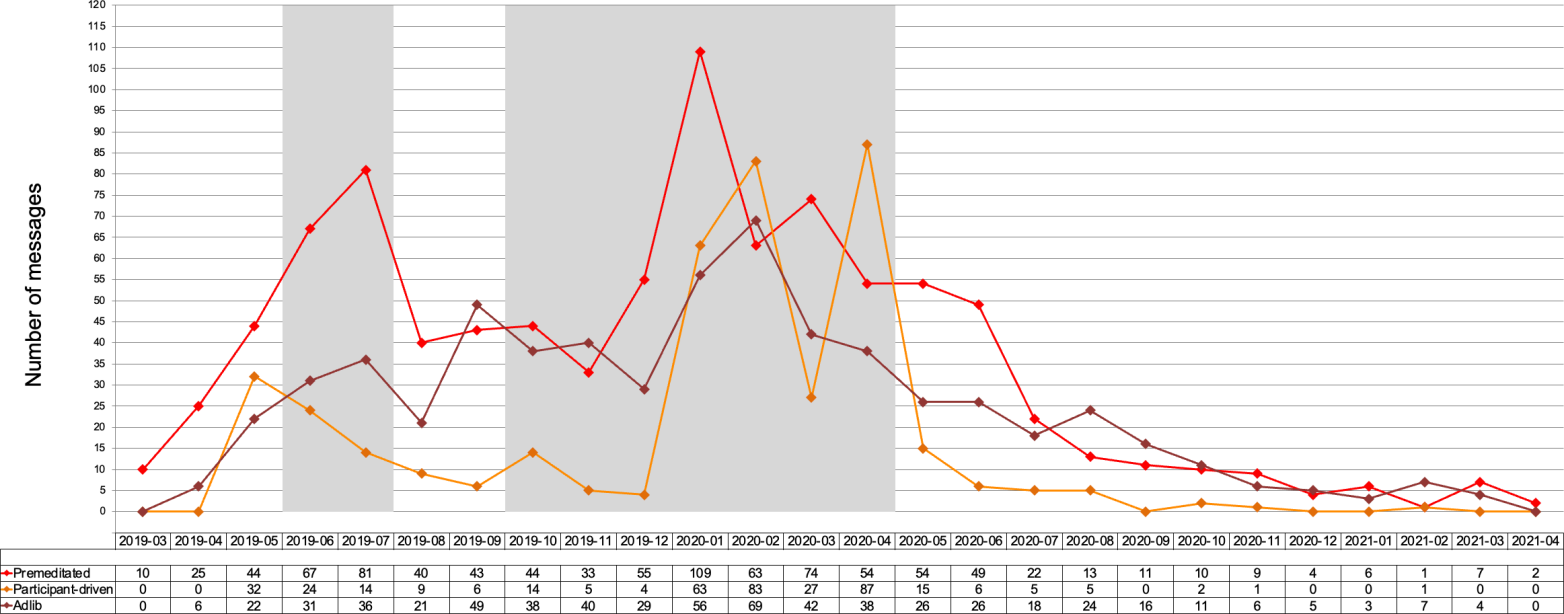
Distribution of messaging type on the Addiction Comprehensive Health Enhancement Support System message board. A-CHESS: Addiction Comprehensive Health Enhancement Support System.

The discourse analysis on the app usage identified 3 coexisting message structures: premeditated and adlib messaging (both driven by case managers and research staff) and participant-driven messaging. Initially, research staff and case managers implemented a planned and structured approach to communication by posting premeditated messaging, using predetermined topics, scheduled posts, and automated motivational messages within the app. Adlib messaging emerged approximately 1 month into the study, with research staff leading the communication. Compared to the premeditated messaging, the staff began incentivizing participant engagement and shared personalized stories to encourage active participation. In the participant-driven phase of messaging, participants actively shaped the app’s interaction patterns, taking ownership and initiating discussions, fostering autonomy and creativity within the community by sharing unique messages alongside and inspired by the premeditated messages of the research staff. Although the premeditated phase occurred at the start of the app use and dominated through the 14 months of app usage, the messaging had transitions between phases, with elements of each phase coexisting or overlapping with others (Figure 1). Figure 1 shows the monthly message types on the A-CHESS app message board among people living with HIV and SUDs from 2019 to 2021.

### Premeditated Messaging

The research staff followed a structured approach by planning and scheduling their posts in advance. They created a weekly schedule to outline the topics they intended to cover on the app, encompassing various subjects such as raising awareness about current issues, providing news resources, and sharing health-related information.

#### Premeditated Messaging: Thought of the Day

The A-CHESS app includes automated “Thought of the Day” (TOTD) posts shared with participants daily at 4:00 PM, featuring short motivational quotes attributed to their authors. One participant started responding to these TOTD messages in the first month of the study by stating their agreement with the TOTD message. A month later, other participants started to engage in the same behavior and provided interpretations of the TOTD.

##### Aligning With TOTD

When participants responded to the TOTD posts, some showed alignment with the posts by expressing an agreement or endorsement using words such as “yes” or “I agree.” In the example below, JAXSTORM responded to the TOTD post by simply agreeing that the TOTD is true. The response from JAXSTORM demonstrates a brief affirmation of the TOTD without further elaboration or explanation (Textbox 1).

Textbox 1. **Example of participant alignment with a “Thought of the Day” post through brief affirmation.**

**A-CHESS App| April 17, 2020, 04:00 PM**

Thought of the Day–“The sleeper gets nothing but the dream.”

**JAXSTORM | April 17, 2020, 06:19 PM**

Yes that is true.

The TOTD uses the metaphor of sleep and dreams to represent a state of inaction or passivity in pursuing one’s goals. JAXSTORM uses the word “true” to not only agree but signal that the message holds truth. Similar alignments to TOTD show participants agreeing with the messages, usually using “yes” and rarely questioning, disagreeing, or offering further detail to align with the TOTD posts.

##### Interpreting TOTD

Participants interpret the TOTD messages by sharing a personalized interpretation of what they mean and what the message tries to communicate to them or others (Textbox 2).

Textbox 2. **Example of a participant’s personalized interpretation of a “Thought of the Day” message.**

**A-CHESS App| May 05, 2020, 04:00 PM**

Thought of the Day–Today I have the courage to look without fear at what needs to be changed in my life.

**AsherTwilight18 | May 05, 2020, 07:51 PM**

Don’t be afraid to change whatever is holding you back.

AsherTwilight18 shares an interpretation encouraging others to embrace change and not fear what might hold them back, interpreting the TOTD message as a call for courage to make changes people fear. While the TOTD included a personal message using an “I” statement, the participant’s interpretation uses “u,” which frames the interpretation as a message to others rather than oneself. AsherTwilight18 changes the message from an “I” statement (with” have the courage”) to a more directive statement (“don’t be afraid”). AsherTwilight18 also interprets what needs to be changed as “whatever holds a person back,” going further than the initial quote by suggesting that a change is needed because something is holding people back.

##### Applying TOTD to Self

When participants apply TOTD posts to themselves, they often connect the message and personal experiences to their addiction and sobriety. This personal application of TOTD posts allows participants to find meaning and relevance in the TOTD messages based on their experiences. The TOTD example below shows a participant responding to a message about a behavior change (Textbox 3).

Textbox 3. **Example of a participant applying a “Thought of the Day” message to personal behavior change.**

**A-CHESS App| March 05, 2020, 04:00 PM**

Thought of the Day–The change of one simple behavior can affect other behaviors and thus change many things. Jean Baer

**Allen18 | March 03, 2020, 08:05 PM**

I don’t drug today. Instead I get good sleep and can achieve more positive outcomes.

Allen18 identified their “change of one simple behavior” as not doing drugs. They used “I” statements to apply the message of the TOTD to themselves in their present day. While the TOTD is about change, the participant uses “instead” to show their behavior change—getting good sleep instead of doing drugs. Marking that sleep is “good” also shows the effect of not doing drugs on another behavior (sleep), as the change of one behavior affecting another is suggested in the original quote.

### Responses Combining Aligning, Applying, and Interpreting TOTD

In posts with several responses, 1 participant may apply the message within the post by connecting it to their personal experiences, behaviors, or challenges. At the same time, other participants may interpret the TOTD by offering their insights, perspectives, or alternative ways of understanding the message. These multiple functions within a single response demonstrate the richness and complexity of participant engagement with the TOTD. Participants bring their own experiences, interpretations, and reflections to the message, resulting in multifaceted interactions. Here is an example of these occurring responses to the same TOTD post (Textbox 4).

Textbox 4. **Example of participant responses combining alignment, interpretation, and application of a “Thought of the Day” message.**

**A-CHESS App| April 29, 2020, 04:00 PM**

Thought of the Day–God Listens to Knee Mail.

**PsycheLark | April 30, 2020, 12:00 PM**

Simply to pray everyday.

**Luna12 | April 30, 2020, 12:39 PM**

He Sure Does.

**AsherTwilight18 | May 1, 2020, 12:26 AM**

Bend your knees.

**JAXSTORM | May 1, 2020, 2:55 PM**

God listens to all his children.

In this thread, 4 participants responded uniquely to the TOTD post about God listening to “knee mail,” a metaphor for prayer, an example of using recovery language. PsycheLark interpreted the TOTD by explaining that it encourages praying every day. Their response focused on understanding the message conveyed by the TOTD. Luna12, on the other hand, aligned with the TOTD by agreeing that God, “He,” does indeed listen. Their response showed support for the sentiment expressed in the TOTD. The response from AsherTwilight18 was brief but still connected to the TOTD by suggesting to “bend your knees,” reinforcing the concept of prayer. Finally, JAXSTORM also interpreted the TOTD by stating that God listens to “all his children.” Their response aligned with the idea of God’s attentiveness to prayers. These participants, without directly interacting with one another, demonstrated different ways of engaging with the TOTD, including interpretation, alignment, and brief expressions related to the message of the TOTD.

#### Premeditated Messaging: News

The research staff shared the news to disseminate information about what was happening in the study setting or noteworthy happenings relevant to the study group participants. Usually, the news posts were approximately a paragraph (longer than TOTD) and had external links providing the news source and more information about the news (Table S1 in Multimedia Appendix 1). The news posts focused on recovery, health care topics, or recovery news. In contrast to the active engagement observed with TOTD posts, the news shared in the message thread did not elicit any responses from the participants. In addition to news reports, case managers and research staff raised awareness on issues similar to news but corresponded to months dedicated to these efforts, such as stress awareness month.

#### Premeditated Messaging: Raising Awareness

The research staff intentionally posted messages to raise awareness on relevant topics for the study participants. These posts focused on stress awareness, sexual assault awareness, important historical days related to HIV, and other health-related subjects. In addition, awareness included appreciation for health care professionals, Drug Enforcement Administration (DEA) drug-take-back days, mental health awareness days, overdose awareness days, and significant events such as World AIDS Day on December 1. These topics were selected to share pertinent news and actively engage participants in discussing their SUD or HIV status.

Participants responded more frequently to the awareness topics compared to the news posts. The research staff’s efforts to tailor the information to the participants’ experiences and interests likely contributed to their engagement and willingness to participate in discussions on these topics. Table S1 in Multimedia Appendix 1 shows an example of an awareness message shared by a case manager about the HIV “U=U campaign” sharing an external link to an American Medical Association message about HIV transmissibility and viral load suppression.

Most of the premeditated messages did not attract engagement from participants. To address the low engagement in the message board, the research staff and case managers posted more adlib messages that followed a similar framework as the premeditated messages but included overt incentives and direct questions to engage participants actively. By incorporating incentives and asking specific questions, the staff encouraged participants to actively participate in discussions about the awareness topics and increase engagement with the posts.

### Adlib Messaging

The research staff and case managers introduced adlib messages to increase participant engagement. Some incorporated incentives and encouraged active participation. They started incentivizing engagement by offering rewards or benefits to participants who actively participated in discussions. In addition, the staff used various techniques to encourage participation, such as sharing polls on different topics of interest.

Table S2 in Multimedia Appendix 1 describes messages in the spontaneous phase of the study, characterized by research staff and case managers sharing entertaining messages, destigmatizing struggles, and incentivizing participant engagement.

#### Adlib Messaging: Sharing Entertainment

Research staff and case managers began sharing entertainment posts to engage participants. These entertainment posts ranged from jokes to memes and riddles. While some of the adlib entertainment received engagement, the research staff further simplified the means of engagement with these messages by creating polls where participants could select an answer option. These polls were introduced 6 months into the study, coinciding with when engagement on the app saw a decline, as seen in August in Figure 1. The polls were well received, and the research staff incentivized responses to these polls to drive engagement. An example of the incentivized polls is in Table S2 in Multimedia Appendix 1.

Other entertainment posts followed similar message patterns as the premeditated message where case managers raised awareness about fun topics such as “National Chocolate Covered Anything Day!" They provided external links to resources about chocolate, similar to when they raised awareness about HIV and SUD topics when posting premeditated messages.

In addition to entertaining messages, case managers approached adlib messaging by including more personalized stories about their everyday struggles to normalize difficult life experiences.

#### Adlib Messaging: Normalizing Struggles

Case managers openly shared various topics, including their weaknesses and challenges. This shift in messaging type showed case managers revealing more about themselves, sharing personal stories about pet loss, lack of motivation, living with pain, and other relatable experiences. These personal messages were also followed by external resources or directed questions to engage participants about their experiences that may be similar to those of the case managers. An example in Table S2 in Multimedia Appendix 1 shows a case manager sharing their pain experiences and providing resources for pain relief.

These adlib entertainment and personalized posts allowed others to engage with the staff’s posts. However, only participants who typically respond to posts engaged with those posts. However, posts that incentivized participant engagement received responses from participants who had never responded to a message on the board.

#### Adlib Messaging: Incentivizing Participant Engagement

The research staff members occasionally introduced incentives such as challenges or contests to encourage further participation. These incentives could include gift cards, recognition, or tangible or symbolic appreciation. Incorporating these incentives motivated participants to engage with the app actively. In the example below, a user disagrees with the crowned winner of a weekly challenge.

Example interaction (Table S3 in Multimedia Appendix 1) shows the case manager named a winner and invited feedback as commonplace with the adlib messaging. NovaBliss challenges the announcement of the winner in several ways. First, by asking who the participant is (displaying their perceived lack of participation in the app); second, by asking about eligibility; third, by more overtly disputing that the winner had engaged in discussion with sensory evidence (“I didn’t even see”); fourth, by asking about how the winner is selected; and fifth, by sharing their feelings about who should have won. This shows the participant’s comfort in confronting the staff and standing up for other group members. Case manager also uses exclamation marks in their explanation, which suggests a friendlier tone that may be used to resolve the conflict [27]. Although NovaBliss was not satisfied with the response to their question, Luna12 stepped in and shared their appreciation, starting with “aww” as a description of how they felt about NovaBliss's gesture.

Although this post first declared the winner of a contest incentivizing participant engagement, the responses work through conflict negotiation, which often demonstrates togetherness and allyship in communities. This interaction also signals participants’ knowledge of their peers’ contributions, especially the key contributors. Key contributors such as Luna12 often responded to the adlib and premeditated messages. However, they also adapted those messaging styles to create posts relatable to their experiences and other participants. These messaging types are described as participant-driven messaging.

#### Participant-Driven Messaging

During this phase, participants mirrored messaging styles the research staff and case managers created while tailoring them to their sobriety and HIV needs. As described in Table S2 in Multimedia Appendix 1, participants adopted the TOTD posts as a model and provided similar messaging that pertained to their journey toward sobriety. Participants also applied the adlib messaging approach by crafting their own polls and sharing their entertainment content. Finally, participants used the message board beyond modeling the type of messages posted by the research staff, including case managers to seek advice, share their substance use experiences, and address psychosocial needs, including dating and local activities. These messaging types signify the emergence of an autonomous and engaged community where participants take ownership of the app, fostering a supportive network and sharing valuable resources.

#### Participant-Driven Messaging: Model Following

Participants followed the model created by research staff similar to the premeditated and adlib messages that involved sharing TOTD, sharing entertainment, and creating polls to drive engagement. However, when participants created their initial contributions, they tailored them to things specific to their experiences and recovery. For example, one participant started sharing messages similar to the TOTD. However, their messages were daily using Alcoholics Anonymous literature.

#### Participant-Driven Messaging: Seeking Tailored Support

Participants who had limited engagement on the app began seeking personalized support. They reached out through messages to ask specific questions, seeking guidance on various topics such as dating, substance use, COVID-19 protocols, or legal assistance. In these instances, both case managers and peers played a role in responding to these inquiries.

Table S2 in Multimedia Appendix 1 shows a contrast between how research staff interacted with participants when they sought advice, which differs from when participants interacted with one another.

London57 discusses their potential need for legal assistance soon but expresses uncertainty about the exact circumstances. Research Staff shares a link to a legal aid service without further inquiring about the situation of London57. In contrast, when participants engaged with one another, they tended to ask more questions before offering support. This approach may serve multiple purposes, such as a better understanding of how they can provide assistance or getting to know the individual better to establish camaraderie. Additionally, participants started sharing a desire to include additional features in the app, which differs from advice where they are asking for support.

#### Participant-Driven Messaging: Adapting the App Use

Adapting the use of the app involved a few participants who took the initiative to create message types specific to their recovery and medication use. Some participants shared their profiles for dating, using the app as a platform to meet someone, similar to a dating app. In the example below, Luna12 starts medication check-in, adding that checking on one another is what the group is for (Textbox 5).

Textbox 5. **Example of participant-driven adaptation of app use through a medication adherence check-in initiative.**

**Luna12|April 05, 2020 05:54 PM**

2020-04-05 17:54:33 UTC

Check in: Hi everyone. Let’s start doing a I took my meds today check-in. Starting tomorrow. How about it?

**Case Manager|April 06, 2020 07:19 AM**

This is an awesome idea!! Thank you for organizing this.

**Luna12|April 06, 2020 11:49 AM**

That’s what this group is about. We need to check on each other.

In this example, Luna12 introduces an idea centered on a daily behavior check-in. They also seek direct feedback from other participants. The case manager uses exclamation marks to signal their enthusiasm and support for the idea proposed by Luna12. However, after several attempts to start medication check-ins, there was a lack of engagement and follow-up; thus, Luna12 discontinued the medication check-in.

Including new features shows participants focus on doing something daily on the app and how they perceive the group’s use. As such, participants also perceived the group as a place they could plan to meet and interact with others with whom they shared similar lived experiences.

#### Participant-Driven Messaging: Community-Building Beyond the App

Participants wanted to connect outside the app, indicating a need for an in-person community. They took the initiative to plan meetups and events, sharing their phone numbers and discussing potential activities in their local areas. This demonstrated their eagerness to foster real-life connections and engage in social interactions beyond the digital realm of the app. In the example below, SpiritSeeker shares their number to ask participants to connect with them (Textbox 6).

Textbox 6. **Example of participant-driven community-building through sharing contact information to connect beyond the app.**

**SpiritSeeker|January 20, 2020 01:36 PM**

Need a ear,That’s Me. You you need a ear,some one to listen,I here,M/ number is [ phone number] call any time,I care.

**Luna12|January 20, 2020 03:45 PM**

Thank you.

SpiritSeeker identifies themselves as a caring person. Although they do not contribute frequently with other participants, they asked to be reached anytime outside the app. Gratitude expressed by Luna12 and the case manager shows an appreciation for the gesture of SpiritSeeker. Other participants also offered to contact them and connect outside the app, showing community building moving from the mobile app to in-person meetings.

## Discussion

### Principal Findings

Our study used computer-mediated discourse analysis to investigate the formation of a virtual community among individuals living with HIV and SUD on the A-CHESS mobile health app. Using CMDA, we identified three different types of messaging: premeditated, adlib, and participant-driven, operating within the A-CHESS message board to shape the evolving norms, tone, and purpose of the community. Of particular interest is how the discourse evolved over time: staff initiated communication with structured posts and motivational content while participants progressively mirrored, repurposed, and transformed those formats to address their own needs, including support around recovery, medication use, and social connection. This study is one of the few to examine how virtual community formation unfolds within a mobile health platform, and to our knowledge, the first to do so among individuals living with both HIV and SUD. Rather than focusing solely on usage metrics or predefined support categories, our approach surfaces the evolving discursive norms, message structures, and participant adaptations that shaped the A-CHESS message board. We offer a novel perspective on how meaning, alignment, and ownership emerged through digital peer interaction in a multiply marginalized population for virtual community formation.

While there is no universally agreed-upon definition of an online community, it is often characterized as individuals engaging in virtual interactions, guided by established norms and policies, and supported by technology [28,29]. Notably, the formation of the A-CHESS virtual community was characterized by the active involvement of case managers and staff in driving participant engagement through adapted messaging styles, aligning with previous research emphasizing the role of community moderators in facilitating interaction [30]These findings suggest opportunities for future research to examine how different roles, such as peer navigators or moderators or patient navigation services, may support engagement and clinical outcomes when integrated into mobile app based support interventions. Of note, participants tended to mirror the behaviors of staff and their peers. The messaging types showed, particularly in adlib messaging, a shared understanding of the established norms of message response, consistent with the influence of social dynamics within online communities [31]. Participants mirrored sharing approaches that were similar to engaging with motivation TOTD and also used polls for engagement. Participants also demonstrated agency by shaping the app to meet their individual support needs, showcasing a sense of empowerment and ownership within the community.

Active engagement and interaction among members are vital to fostering online community relationships [32]. Our 26-month study revealed interesting trends in participant engagement and interaction. While only a subset of participants interacted with messaging, this aligns with research on app engagement that has shown engagement frequency to be limited to a few individuals [31,33]. It is important to acknowledge that other participants may have engaged passively, commonly referred to as “lurking” by reading and benefiting from message board content without posting. As noted in the literature, the 90-9-1 rule describes this dynamic, where approximately 90% of users are “lurkers,” 9% contribute occasionally, and just 1% are highly active [34]. This form of invisible engagement is not captured through message analysis but may still reflect meaningful interaction with the app and community [35]. Within A-CHESS, active app participation peaked between months 10 and 14 but experienced a decline from month 18, which was 9 months before the study’s conclusion. It is worth noting that the COVID-19 lockdown started during month 14, which could explain the increase in participant-driven messages during March 2020. Nonetheless, our study’s pattern of engagement differed from findings in a study on an HIV message board, where post numbers peaked in the first 6 months, decreased between months 6 and 12, and then increased again between months 18 and 24 [10]. Additionally, our study identified a gradual increase in participant-driven interaction during the first year, indicating a shift toward seeking tailored support and taking ownership of the conversations within the app.

The premeditated motivational messages in our study received the highest level of interaction from all study participants compared to all other messaging types, underscoring the significance of motivational messages within the virtual community. This finding is consistent with a study focused on improving engagement in HIV care, where participants expressed enthusiasm for receiving motivational messages [36]. Our study participants also demonstrated a unique way of responding by applying, aligning, or interpreting the messages. The alignment observed in virtual community formation is often seen in agreement or shared perspectives among group members [37], but in this case, participants aligned themselves with automated messages. Mobile health apps can continue integrating motivational messages to enhance user engagement and encourage users to interact with and respond to such messages actively.

It is worth noting that premeditated news and awareness messages in the A-CHESS virtual community did not generate significant interaction from participants. This lack of engagement can be attributed to the primary focus of participants in recovery groups on addressing feelings of loneliness and uncertainty, as observed in previous studies on support-seeking behaviors in individuals with SUDs [38]. This finding aligns with results from an HIV online support group where messages providing jokes and pleasantries were posted as often as messages providing emotional support [39]. The discrepancy in engagement underscores the significance of incorporating varied message types that cater to community members’ diverse needs and preferences. Despite different feelings and emotions, our study shows that attending to these emotions is beneficial compared to sharing news or raising awareness.

The adlib messages from case managers played a significant role in fostering virtual community formation within the A-CHESS app. Research studies have highlighted that members of online communities are more likely to form relationships when they have opportunities for self-disclosure and learning about each other [31]. By personalizing their messages, case managers allowed participants to gain insights into the staff and case managers. While incentives encouraged participation, they also introduced tension, as some participants perceived the reward structure as unfair or misunderstood. These moments suggest a need for further research on how extrinsic motivators shape digital peer dynamics, especially within marginalized populations.

In the participant-driven message types observed in our study, key participants played an important role in initiating and maintaining interactions among participants, including responding to questions. These findings align with previous research on message board usage among people living with HIV, which also identified the influential role of a few individuals in driving engagement [14]. Our study highlighted the unique use of the message board by participants seeking support and guidance regarding dating, reflecting the ongoing relevance of discussions about intimacy and relationships within online communities for individuals living with HIV [40]. Additionally, our findings revealed that initial online connections often developed into offline meetups, reflecting the establishment of deeper relationships within the community.

We also observed differences in how staff and case managers interacted with participants compared to how participants interacted with each other within the virtual community. Participants were more likely to ask follow-up questions, while research staff primarily provided informational support. The act of asking questions is valued in online communication as it promotes efficiency, purposefulness, and clear understanding [41], fostering interaction within these communities. Additionally, studies have indicated that responses to questions and requests for advice from participants are often met with emotional support in online environments [42].

While our study focused on the A-CHESS virtual community, it is worth noting that messaging app usage has often been associated with younger, white individuals of higher socioeconomic status [43]. However, participants contributing to the A-CHESS message board were predominantly older, Black, and unemployed. This demographic disparity highlights the importance of exploring the specific types of social support individuals from different racial backgrounds seek within mobile health apps. Future research could examine the preferences and needs of diverse populations, informing the development of tailored apps that enhance engagement and user experience for individuals from various racial backgrounds.

Online communities thrive when members actively interact, maintain the community infrastructure, share new and updated information, and provide social and emotional support to fellow members [31,44]. In the context of the A-CHESS app, the collaborative efforts of case managers, research staff, and engaged participants collectively contribute to forming and sustaining the virtual community for the study duration. We recommend that future mobile health interventions for people living with HIV and SUD support virtual community formation by integrating diverse message types and fostering participant agency. Facilitating participant-driven discourse can help individuals tailor the app to their needs, strengthening feelings of ownership and peer connection. Future app design and implementation strategies should therefore consider not only incentivizing engagement but also supporting the organic development of peer-to-peer communication, including opportunities for participants to shape content and norms. Further research could explore how different messaging types influence recovery outcomes across alcohol, opioid, stimulat use, as well as HIV treatment engagement , and how nonvisible engagement (such as passive participation) contributes to community cohesion and support.

### Limitations

Our study has several limitations that should be acknowledged. First, while discourse analysis provides insights into participants’ expressed intentions and behaviors, it does not provide direct access to their thoughts and motivations, limiting our ability to determine the intended behaviors behind each message posted. Additionally, the message board as the source of discourse limits the ability to explore how participants navigated certain emergent dynamics, such as minor conflict or unacknowledged support requests, beyond what was expressed in writing within the board. Second, the generalizability of our findings may be limited due to the specific population under study. The A-CHESS app was distributed to individuals living with HIV and SUD in a midwestern state, and the unique characteristics and context of this population may influence their engagement and interactions within the virtual community.

### Conclusion

In conclusion, we identified three message types shaping interaction on the A-CHESS mobile app: premeditated, ad lib, and participant-driven messages. Staff-initiated messaging contributed to patterns of engagement observed on the message board, while participant-driven posts reflected peer connection and shared recovery experiences relevant to SUD in people living with HIV. Our research staff and case managers drove participant engagement through premeditated and adlib messaging. Participants contributed tailored messages, demonstrating their interest in engaging and connecting with peers. When developing mobile apps for social support, it is important to identify the role of those leading the platform in driving engagement and to adapt engagement strategies to meet participants’ needs.

## Supplementary material

10.2196/66564 Multimedia Appendix 1 Addiction Comprehensive Health Enhancement Support System message board threads.

10.2196/66564 Checklist 1 Standards for Reporting Qualitative Research (SRQR) checklist.

## Abbreviations

A-CHESS: Addiction Comprehensive Health Enhancement Support System
CMDA: computer-mediated discourse analysis
DEA: Drug Enforcement Administration
PWUD: people who use drugs
SRQR: Standards for Reporting Qualitative Research
SUD: substance use disorder
TOTD: Thought of the Day
