# Supplementary material for: Support Community Formation on a Mobile App for People Living With HIV and Substance Use Disorder: A Computer-Mediated Discourse Analysis
Source: JMIR Form Res. 2026 Jan 15;10:e66564. doi: 10.2196/66564 (PMC12807402; doi:10.2196/66564)
Supplement: Multimedia Appendix 1 [file formative-v10-e66564-s001.docx]

Supplement 1: Support Community Formation on a Mobile App for People Living with HIV and Substance Use Disorder: A Computer-Mediated Discourse Analysis

**Supplement Overview**
This supplement presents the three message types (premeditated, ad lib, and participant-driven) identified through computer-mediated discourse analysis (CMDA) of message board posts on the A-CHESS (Addiction Comprehensive Health Enhancement Support System) mobile application. The A-CHESS message board was used by individuals with comorbid HIV and substance use disorder, and messages included in these tables were posted between April 2019 and May 2021. The tables provide examples of messages posted by case managers, research staff, and participants, along with representative responses and observations related to engagement patterns, providing insight into how a support community is formed through interaction within a mobile health application.

Table of Contents

[Table S1: Premeditated Message Types, Sample Posts, and Responses on the A-CHESS Message Board 2](#_Toc218852031)

[Table S2: Ad Lib Staff Message Types, Sample Posts, and Responses on the A-CHESS Message Board 5](#_Toc218852032)

[Table S3: Participant-Driven Message Types, Sample Posts, and Responses on the A-CHESS Message Board 10](#_Toc218852033)

# **Table S1: Premeditated Message Types, Sample Posts, and Responses on the A-CHESS Message Board**

| Message types | Description | Sample of initial and response messages | Engagement summary |
| --- | --- | --- | --- |
| Thought of the day messages | The A-CHESS app distributed automated messages. These were motivational messages that often included an inspirational message and the author of the message. Messages were randomly generated daily, and participants responded by aligning, applying, or interpreting the messages to themselves and other participants on the app. | **[Initiation post]**  **A-CHESS App\| March 12, 2019, 04:00 pm**  Thought of the Day – "A wise man gets more use from his enemies than a fool does from his friends. "  **[Response posts]**  **JAXSTORM\| March 13, 2020, 07:14 am**  >That is true all day.  **LunaSmiles\| March 13, 2020, 08:34am**  >To me it means sometimes you need everyone help getting thought the day  **greensquirrel4551\| March 13, 2020, 10:26 am**  >To me the thought of the day means choice your friends wisely  **MoonlitWander\| March 13, 2020, 02:06pm**  >My mom use to say that to me with I was younger.  **AsherTwilight18 \| March 13, 2020, 1:28p**  >A fool and his freind will be just that fools | Most of the messages in the app were responses to TOTD posts. Participants started to follow one participant's engagement with the TOTD without explicit instructions on whether or how to respond, highlighting the development of community norms within the online space. |
| News messages | ACHESS case managers shared the HIV and SUD news with participants. Other news topics included  current affairs, such as elections, and later included COVID-19. | [**Initiation post]**  **Case Manager\| April 09, 2019 11:32 am**  > Hi All-The first kidney transplant from a person living with HIV was given to another person who is living with HIV. I think this is awesome and I am happy to hear of this new milestone happening! What are your thoughts on this? [ Link] Happy Tuesday, [Name]  **[No responses]** | Approximately 150 messages posted on the app were news contents. Case managers posted news messages. However, participants did not interact or engage with the news. |
| Awareness messages | Research staff and case managers also raised awareness on important HIV and drug use topics/ | [**Initiation post]**  **Case manager\| April 16, 2019, 09:52 am**  >Hey everyone,Have you heard of the U=U campaign? Undetectable Equals Untransmittable. U=U signifies that individuals with HIV who receive antiretroviral therapy (ART) and have achieved and maintained an undetectable viral load cannot sexually transmit the virus to others.  **[No responses]** | Approximately 148 posted on the board were related to raising awareness. Similarly, participants did not engage with messages that were raising awareness and often only acknowledged the messages. |

# **Table S2: Ad Lib Staff Message Types, Sample Posts, and Responses on the A-CHESS Message Board**

| Message types | Description | Sample | Engagement summary |
| --- | --- | --- | --- |
| Sharing entertainment | Research staff and case managers shared entertaining content, such as funny anecdotes, jokes, memes, or interesting articles, to add a light-hearted and entertaining aspect to the discussions. | **[Initiation post]**  **Research Staff\| December 11, 2019, 01:51 pm**  "How do you make an octopus laugh?  With ten-tickles!"  **[Response posts]**  **Luna12\| January 08, 2019, 03:58 pm**   \| Hahahaha  **OrionAdventures\| January 31, 2019, 10:46 am** \| 2020-01-08 15:38:00 UTC \| Luna12 \| \| --- \| --- \| --- \| \| Thanks for the laugh  **WildSoul\| February 01, 2019, 04:25 pm** \| 2020-01-31 10:46:34 UTC \| OrionAdventures \| \| 💖I thought by Tenkling...lol💖 \| 2020-02-01 16:25:02 UTC \| WildSoul \|   **[Initiation post]**  **Research Staff\| April 08, 2020, 01:47pm**  What's your favorite non-alcoholic beverage? Describe it or tell us why in the comments.  A) Coffee B) Black tea, hot or iced C) Juice D) Water E) Pop or soda F) Herbal infusions G) Other?  Coffee is my morning regular. It's more of a habit than a favorite.  **[Response posts]**  **Luna12\| April 08, 2020, 11:59pm**  I love black tea. Now that's my favorite. I can drink it iced with sugar or hot .  **NovaBliss\| April 10, 2020, 08:36 am**  I love sprite its my favorite and it has no caffeine in it. | Participants engaged with the entertainment by laughing, using emojis, or sharing their jokes. Unlike TOTD posts, the responses were not immediate and sometimes occurred after several weeks. Additionally, various participants responded to the entertainment posts. |
| Normalizing struggles | Before sharing resources with the group, the research staff and case managers openly discussed and shared their struggles or challenges related to different health-related topics. These differed from the news or awareness in the premeditated phase as staff started the posts by sharing their personal life before sharing the health information and resources. | [**Initiation post]**  **Case Manager\| April 09, 2019, 11:32 am**  Back in April of this year I started experiencing lower back pain. I wasn't sure what the cause of it was, but for over a week I rested my back thinking that was the only thing that would help it heal. After that week, it felt worse! I started working out again and to my surprise, it started to feel better. I was not sure what to make of all of it, until I saw this article on NPR about how you can establish pain relief by exercising. What exercise works for one, might not work for another person. Click [link] read more. What do you all think about this? Has it worked or not worked for any of you?  Happy Friday ✨  **[No responses]** | Participants did not engage with posts that normalize struggles. The few participants that frequently engage with messages on the board acknowledged some of the posts. However, participants did not engage with part of the posts where staff shared their personal lives. |
| Incentivizing participant engagement | The research team actively encouraged and motivated others to participate in discussions, surveys, or other activities within the study. They offered rewards, incentives, or recognition to promote engagement and ensure active participation from other participants. | **[Initiating post]**  **Case manager\| June 09, 2020, 11:56 am**  "What does the "E" stand for in the name of the American restaurant chain Chuck E. Cheese?  Please message Research or I with your answer! The winner will be picked on  **[Response post]**  **StarJR\| June 10, 2020, 01:42 pm**  Entertainment | Posts that incentivize participant engagement received the most responses to the adlib messages. Participants responded to posts asking riddles and offering incentives for their engagement. |

# **Table S3: Participant-Driven Message Types, Sample Posts, and Responses on the A-CHESS Message Board**

| Message types | Description | Sample | Engagement Summary |
| --- | --- | --- | --- |
| Model following | Participants followed the model by research staff and case managers by sharing entertainment messages, creating their poll, and TOTD messages. | [**Initiation post]**  **WildSoul1\|February 13, 2020 01:14 pm**  Supebowl Poll Questions and Rating  "What was your favorite Super Bowl commerical? Did you watch the Superbowl for the commercials or the halftime show?  I rate the Doritos commerical with the horses and Lil Nas and the dancing mastache a 5  I have no ratings for the halftime show. It was very entertaining as usual"  **[ Six responses omitted from this thread]** | Participants engaged with posts that modeled the research and case managers message types. Research staff also contributed to the messages, further encouraging engagement. |
| Seeking tailored support | Participants asked for information about issues concerning their lives, such as sobriety and legal advice, and sought updates about current issues, such as COVID-19 updates. | [**Initiation post]**  **NovaBliss\|April 10, 2020 08:41 am**  So my daughter is 19 and she is having a baby. I'm a little upset about it, but I'm excited to see my grandbaby. I guess I wish you would have waited a little till she's a little older. At 19 you don't really know how to be a mom I know because I was a mom I got pregnant when I was 19 I had her at 20 and now she's going to be 22. Thank you for listening can anybody give me some advice on how to just accept it because right now I'm a little upset  [**Response posts]**  **Case Manager\|April 10, 2020, 02:02 pm**  Wow, that is a lot to process! Thank you for sharing with us. I hear your concerns about your daughter getting pregnant so young, but I'm glad you're excited to meet you grandchild. There are a few resources out there for expecting women so make sure to take care of yourself first and be there for your daughter as much as you can.  **Luna12\|April 11, 2020 08:44am**  You're not alone and your story sounds better than mine. | Like the modeled posts, research staff engaged with the messages seeking advice. Participants also contributed and asked insightful questions, often requiring someone seeking advice to share more information about their issues. |
| Adapting the app use | Participants started trends in the app to add additional services or features that they perceived benefits, such as sharing their profiles for dating and conducting medication check-ins. | **London57\|July 04, 2020 07:37 am**  Legal help: I recently discovered I may be needing legal assistance or representation in the near future. I know that (clinic name) offers some assistance but I don't know how much they are capable of handling. I don't have the funds to hire an attorney. Does anyone have any suggestions or know of anyone that might offer help at a lower cost?  **Research Staff\|July 06, 2020, 04:26** >Here is the [link] to the Legal Aid Society of (City name). Their phone number is (number) Hope they can help you or refer you to a place who can assist you.  **[Initiating Post]**  **SpiritSeeker\|January 24, 2020, 03:56 pm**  Im single 59 years old,did 28 years in prison been out for a year have my life pretty much together want to share it with someone special,lonely,  **Luna12 \|January 25, 2020, 12:00 pm**  What were you in prison for  **SpiritSeeker\|January 25, 2020 03:56 pm**  Negligent homicide and Burglary  **Titan82 \|January 26, 2020 09:39 am**  What happened  **WildSoul\|February 6, 2020 09:39 am**  💞Hello SpiritSeeker...nice to acquaintance you...thank you for being honest and fair to yourself...you are in the right place for support and gaining knowledge...you can Pvt/D.M. if you would like to or message here and I will gladly respond...💞 | Adapting the app was driven by a few participants. The research staff and other participants were receptive and encouraged others to participate. |
| Community-building beyond the app | Participants started sharing their phone numbers and creating events to meet outside of the app. | **TrayTon \|February 23, 2020, 08:06 pm**  Whats good to do in (City name)  **NovaBliss\|February 24, 2020, 10:58 am**  Bowling, sky zone, round 1, boat cruse  **Luna12\|February 24, 2020 01:31 pm**  We're planning a bowling trip if you would like to come | Participants were receptive of finding time to meet outside the app and shared the information with other users who engaged with the messages. |
