# Supplementary material for: Support Community Formation on a Mobile App for People Living With HIV and Substance Use Disorder: A Computer-Mediated Discourse Analysis
Source: JMIR Form Res. 2026 Jan 15;10:e66564. doi: 10.2196/66564 (PMC12807402; doi:10.2196/66564)
Supplement: Checklist 1 [file formative-v10-e66564-s002.docx]

|  | Standards for Reporting Qualitative Research (SRQR) Form | |
| --- | --- | --- |
| <http://www.equator-network.org/reporting-guidelines/srqr/> | | |
| **Reporting Criteria** | | **Page/line no(s).** |
| **Title and abstract** | |  |
| Title - Concise description of the nature and topic of the study Identifying the study as qualitative or indicating the approach (e.g., ethnography, grounded theory) or data collection methods (e.g., interview, focus group) is recommended | | Title Page Clearly identifies the study as qualitative, using "discourse analysis." |
| Abstract - Summary of key elements of the study using the abstract format of the intended publication; typically includes background, purpose, methods, results, and conclusions | | Page 1  Structured abstract with background, purpose, methods, results, and conclusions. |
| **Introduction** | |  |
| Problem formulation - Description and significance of the problem/phenomenon studied; review of relevant theory and empirical work; problem statement | | Page 2  Describes the significance of studying community formation among people with HIV and SUD using a mobile app. |
| Purpose or research question - Purpose of the study and specific objectives or questions | | Page 2  States the study aim: to examine how community is formed on the message board. |
| **Methods** | |  |
| Qualitative approach and research paradigm - Qualitative approach (e.g., ethnography, grounded theory, case study, phenomenology, narrative research) and guiding theory if appropriate; identifying the research paradigm (e.g., postpositivist, constructivist/ interpretivist) is also recommended; rationale | | Pages 2 – 4  Describes use of computer-mediated discourse analysis (CMDA) and constructivist theory. |
| Researcher characteristics and reflexivity - Researchers’ characteristics that may influence the research, including personal attributes, qualifications/experience, relationship with participants, assumptions, and/or presuppositions; potential or actual interaction between researchers’ characteristics and the research questions, approach, methods, results, and/or transferability | | Page 4 |
| Context - Setting/site and salient contextual factors; rationale** | | Page 3  Overview of the ACHESS app including salient factors participant characteristics and overall aim of the mobile app development. |
| Sampling strategy - How and why research participants, documents, or events were selected; criteria for deciding when no further sampling was necessary (e.g., sampling saturation); rationale | | Page 3  All messages over 26 months included; thread and time-based sampling rationale. |
| Ethical issues pertaining to human subjects - Documentation of approval by an appropriate ethics review board and participant consent, or explanation for lack thereof; other confidentiality and data security issues | | Page 4  IRB approval, anonymity via deidentified usernames, consent procedures described. |
| Data collection methods - Types of data collected; details of data collection procedures including (as appropriate) start and stop dates of data collection and analysis, iterative process, triangulation of sources/methods, and modification of procedures in response to evolving study findings; rationale** | | Pages 2 – 3  App message board posts collected over 26 months; no procedure changes noted. |
| Data collection instruments and technologies - Description of instruments (e.g., interview guides, questionnaires) and devices (e.g., audio recorders) used for data collection; if/how the instrument(s) changed over the course of the study | | Page 3  No formal instruments; Excel software used to organize extracted messages. |
| Units of study - Number and relevant characteristics of participants, documents, or events included in the study; level of participation (could be reported in results) | | Page 4 Number of unique posters not listed; participant characteristics described in results. |
| Data processing - Methods for processing data prior to and during analysis, including transcription, data entry, data management and security, verification of data integrity, data coding, and anonymization/de-identification of excerpts | | Page 3  Extraction, anonymization, Excel management, coding process described. |
| Data analysis - Process by which inferences, themes, etc., were identified and developed, including the researchers involved in data analysis; usually references a specific paradigm or approach; rationale | | Page 3  Computer mediated discourse analysis (CMDA) framework, inductive coding, coder roles and discussions described. |
| Techniques to enhance trustworthiness - Techniques to enhance trustworthiness and credibility of data analysis (e.g., member checking, audit trail, triangulation); rationale | | Page 3  Paragraph on rigor and trustworthiness using Lincoln and Guba’s criteria. |
| **Results/findings** | |  |
| Synthesis and interpretation - Main findings (e.g., interpretations, inferences, and themes); might include development of a theory or model, or integration with prior research or theory | | Pages 4–9  Typology and thematic findings presented, including patterns and shifts in mobile application use. |
| Links to empirical data - Evidence (e.g., quotes, field notes, text excerpts, photographs) to substantiate analytic findings | | Pages 4–9 Quotes provided to support each analytic message type, tables used to supplement results described. |
| **Discussion** | |  |
| Integration with prior work, implications, transferability, and contribution(s) to the field - Short summary of main findings; explanation of how findings and conclusions connect to, support, elaborate on, or challenge conclusions of earlier scholarship; discussion of scope of application/generalizability; identification of unique contribution(s) to scholarship in a discipline or field | | Pages 9–11  Discussion connects findings to existing literature and identifies unique contributions. |
| Limitations - Trustworthiness and limitations of findings | | Page 11  Discusses sample representation, generalizability, and app-based constraints. |
| **Other** | |  |
| Conflicts of interest - Potential sources of influence or perceived influence on study conduct and conclusions; how these were managed | | Page 11  Written as instructed by JMIR when none declared |
| Funding - Sources of funding and other support; role of funders in data collection. n, interpretation, and reporting | | Page 11  Funders acknowledged; no role in data interpretation. |

Reference: O'Brien BC, Harris IB, Beckman TJ, Reed DA, Cook DA. Standards for reporting qualitative research: a synthesis of recommendations. Acad Med. 2014;89(9):1245–1251. doi:10.1097/ACM.0000000000000388
